# Reduction of cycles of neoadjuvant chemotherapy for advanced epithelial ovarian, fallopian or primary peritoneal cancer (ROCOCO): study protocol for a phase III randomized controlled trial

**DOI:** 10.1186/s12885-020-06886-2

**Published:** 2020-05-06

**Authors:** Soo Jin Park, Seung-Hyuk Shim, Yong-Il Ji, Sang-Hoon Kwon, Eun Ji Lee, Maria Lee, Suk Joon Chang, Samina Park, Sang Youn Kim, Sung Jong Lee, Jae-Weon Kim, Ju-Won Roh, San Hui Lee, Taejong Song, Hee Seung Kim

**Affiliations:** 1 Department of Obstetrics and Gynecology, Seoul National University College of Medicine, 101 Daehak-Ro Jongno-Gu, Seoul, 110-744 Republic of Korea; 2 Department of Obstetrics and Gynecology, Research Institute of Medical Science, Konkuk University School of Medicine, Seoul, Republic of Korea; 3 Department of Obstetrics and Gynecology, Inje University Haeundae Paik Hospital, Busan, Republic of Korea; 4 Department of Obstetrics and Gynecology, Keimyung University School of Medicine, Daegu, Republic of Korea; 5 Gynecologic Cancer Center, Department of Obstetrics and Gynecology, Ajou University School of Medicine, Suwon, Republic of Korea; 6 Department of Thoracic and Cardiovascular Surgery, Seoul National University Hospital, Seoul National University College of Medicine, Seoul, Republic of Korea; 7 Department of Radiology, Seoul National University College of Medicine, Seoul, Republic of Korea; 8 Department of Obstetrics and Gynecology, Seoul St. Mary’s Hospital, College of Medicine, The Catholic University of Korea, Seoul, Republic of Korea; 9 Department of Obstetrics and Gynecology, Dongguk University Ilsan Hospital, Goyang, Republic of Korea; 10 Department of Obstetrics and Gynecology, Wonju Severance Christian Hospital, Yonsei University College of Medicine, Wonju, Republic of Korea; 11 Department of Obstetrics & Gynecology, Kangbuk Samsung Hospital, Sungkyunkwan University School of Medicine, Seoul, Republic of Korea

**Keywords:** Ovarian, Fallopian, Peritoneal, Cancer, Neoadjuvant chemotherapy, Cycle

## Abstract

**Background:**

Primary debulking surgery (PDS) and adjuvant chemotherapy is the standard treatment for advanced ovarian, fallopian or primary peritoneal cancer. However, neoadjuvant chemotherapy (NAC) followed by interval debulking surgery (IDS) has been introduced as an alternative, showing similar efficacy and decreased postoperative complications compared with PDS. Although there is still no evidence for whether three or four cycles of NAC used clinically could be adequate, reducing one cycle of NAC is expected to remove more visible tumours and thereby improve prognosis. Thus, we proposed with this study to evaluate the efficacy and safety of reducing one cycle of NAC for advanced ovarian, fallopian or primary peritoneal cancer.

**Methods:**

This study is a prospective, multi-centre, open-label, randomized phase III trial. A total of 298 patients with advanced ovarian, fallopian or primary peritoneal cancer will be recruited and randomly assigned to either three (control group) or two cycles of NAC (experimental group). After the NAC, we will conduct IDS with maximal cytoreduction and then administer the remaining three or four cycles for a total of six cycles of adjuvant chemotherapy. The primary end point is progression-free survival, and the secondary end points are time to tumour progression, overall survival, tumour response after NAC, IDS and adjuvant chemotherapy, radiologic investigation after IDS, tumour response by positron emission tomography-computed tomography after NAC, quality of life, adverse events, success rate of optimal cytoreduction, surgical complexity, postoperative complications and safety of IDS. We will assess these factors at screening, at every cycle of chemotherapy, at IDS, after the completion of chemotherapy, every 3 months for the first 2 years after the planned treatment and every 6 months thereafter for 3 years.

**Discussion:**

We hypothesize that reducing one cycle of NAC will contribute to more resection of visible tumours despite 10% reduction of optimal cytoreduction, which could improve survival. Moreover, two cycles of NAC may increase postoperative complications by 5% compared with three cycles, which may be acceptable.

**Trial registration:**

This study has been prospectively registered at ClinicalTrials.gov on Oct. 2nd, 2018 (NCT03693248, URL: https://clinicaltrials.gov/ct2/show/NCT03693248).

## Background

Standard therapy for advanced ovarian, fallopian or primary peritoneal cancer consists of primary debulking surgery (PDS) including maximal cytoreduction and adjuvant chemotherapy using taxanes and platinums. In particular, the size of the residual tumour after maximal debulking surgery is known as the most important prognostic factor of the disease, and the size of residual tumour needed for optimal cytoreduction has become more stringent since 2010, from less than 1 cm to complete resection of all macroscopic tumours [1].

However, the success rate of optimal cytoreduction depends on the extent of tumours and surgical skills [2], and aggressive tumour resection is related to increased risk of postoperative complications in patients with advanced disease who undergo PDS [3]. To overcome these limitations of PDS, investigators have introduced the concept of neoadjuvant chemotherapy (NAC) followed by interval debulking surgery (IDS), and four randomized controlled trials (RCTs) using three or four cycles of NAC were shown to reduce treatment-related morbidity and improve quality of life without worsening prognosis in advanced ovarian, fallopian and primary peritoneal cancer [4–7].

Nevertheless, there has been controversy regarding the appropriate number of NAC cycles. Although some researchers demonstrated no regulation between number of cycles and prognosis [8, 9], four or more cycles of NAC has been reported to decrease survival [10, 11]. In particular, a recent meta-analysis showed that one additional cycle of NAC could decrease survival by 4.1 months, suggesting that increased cycles of NAC might reduce visible tumours to an invisible degree that can cause disease recurrence because the tumour is not fully resected surgically during IDS [12]. Also, a recent trial showed that hyperthermic intraperitoneal chemotherapy (HIPEC) after NAC followed by IDS prolonged progression-free survival (PFS) by 3.5 months and overall survival (OS) by 11.8 months in advanced ovarian cancer compared with NAC followed by IDS alone, suggesting that HIPEC can be helpful in killing invisible tumour cells during IDS after NAC for improved prognosis [13].

Thus, we hypothesized that more visible tumours could be removed by maximal debulking surgery, which can improve prognosis if three cycles of NAC in the clinical setting is reduced to two cycles. To test this hypothesis, we designed an RCT to assess the efficacy and safety of reducing one cycle of NAC for advanced ovarian, fallopian and primary peritoneal cancer.

## Methods

### Objectives

With this study, we aim to compare survival, success rates of optimal cytoreduction, postoperative complications and quality of life between patients treated with three cycles of NAC and those treated with two cycles of NAC for advanced ovarian, fallopian or primary peritoneal cancer.

### Hypothesis

Reduction of one cycle of NAC may improve prognosis by removing more visible tumours during IDS in advanced ovarian, fallopian or primary peritoneal cancer.

### Study design

The current study is a prospective, multi-centre, open-label, randomized phase III trial that will take place in 7 institutions and that has been registered at ClinicalTrials.gov (NCT03693248).

### Setting

The current study will be conducted at the following 7 institutions in Republic of Korea: Seoul National University Hospital; Konkuk University Medical Center; Keimyung University Dongsan Medical Center; Dongguk University Ilsan Hospital; Wonju Severance Christian Hospital; Kangbuk Samsung Hospital; and Inje University Haeundae Paik Hospital.

### Population

Patients with the following inclusion criteria will be enrolled in this study: 1) between 19 and 80 years of age; 2) histologically proven epithelial ovarian, fallopian or primary peritoneal cancer by one of the two following methods: (1) pathologic confirmation of epithelial ovarian, fallopian or primary peritoneal cancer by diagnostic laparoscopy or laparotomy, in particular, if the adnexae look normal in imaging studies, (2) pathologic confirmation of adenocarcinoma originating from the female genital tract by fine needle aspiration or paracentesis with the satisfaction of the four following conditions: ① presence of pelvic or ovarian mass of any size; ② presence of at least 2 cm mass above the pelvic cavity or malignant pleural effusion or metastatic lymph nodes in the cardio-phrenic, internal mammary, mediastinal, para-tracheal, supraclavicular or inguinal area confirmed by imaging; ③ cancer antigen 125 (CA-125, kU/L)/carcinoembryonic antigen (CEA, ng/mL) > 25; ④ no other malignancies in colonoscopy, gastroscopy and mammography performed 6 weeks before randomization if CA-125/CEA is 25 or less; 3) International Federation of Gynecology and Obstetrics (FIGO) stage IIIC to IVB disease, which requires NAC because it is difficult to expect tumours to be removed completely by PDS; 4) World Health Organization (WHO) performance status 0 to 2; 5) normal hematologic, liver and renal function evaluated by the following laboratory tests: (1) white blood cell ≥3000/μl, (2) absolute neutrophil count ≥1500/μl, (3) platelet ≥100 × 10^3^/μl, (4) aspartate aminotransferase ≤100 IU/l, (5) alanine aminotransferase ≤100 IU/l, (6) serum total bilirubin ≤1.5 mg/dl, (7) serum creatinine ≤1.5 mg/dl; 6) absence of psychological, and socioeconomic limitations affecting participation to this study; 7) informed consent prior to registration and randomization in this study.

However, we will exclude patients with the following conditions: 1) diagnosis of metachronous malignancies within 5 years before enrolment; 2) synchronous tumours except follicular or papillary thyroid cancer treated completely with only surgery, and early gastric or colon cancer treated completely with only endoscopic mucosal resection; 3) carcinoma in situ, non-epithelial or borderline tumour in the ovary, fallopian tube and peritoneum; 4) pregnancy; 5) medical conditions affecting prognosis; 6) clinical evidence of brain or leptomeningeal metastasis or bone metastasis; 7) other treatments affecting clinical outcomes during the period of primary treatment including surgery and chemotherapy using paclitaxel and carboplatin (e.g. HIPEC or intraperitoneal chemotherapy); 8) no informed consent for participating in this study.

### Sample size calculation and randomization

We will recruit patients for 3 years and will observe them for 2 years of after enrolment. Based on a recent trial of HIPEC for advanced epithelial ovarian cancer [13], we assume the median PFS in the experimental group to be 14.2 months, whereas the median value of PFS of control group is assumed to be 10.7 months because reducing one cycle of NAC is expected to result in the resection of more visible tumours, showing similar effects to those for HIPEC. When we assumed an exponential distribution, the two-year PFS rates were 30.99 and 21.12% in the experimental and control groups, respectively, and the number of events required to obtain a power of 70% in the single-sided log-rank test at 5% significance was 238, assuming the event risk ratio of the two groups was constant. A total of 298 patients (149 per group) were required when we considered that the number of patients needed for observing 238 events was 270, and that 10% of the target number of patients would be eliminated. We used PASS software (Power analysis and sample size software: http://www.ncss.com) to calculate the sample size for this study.

After confirming of the inclusion and exclusion criteria, patients will be registered at the online system of the Medical Research Collaborating Center at Seoul National University Hospital for randomization. Patients will be randomly assigned to either an experimental or a control group with a 1:1 allocation, with FIGO stage and histology adjusted as stratified factors.

### Interventions

**Fig. 1:**
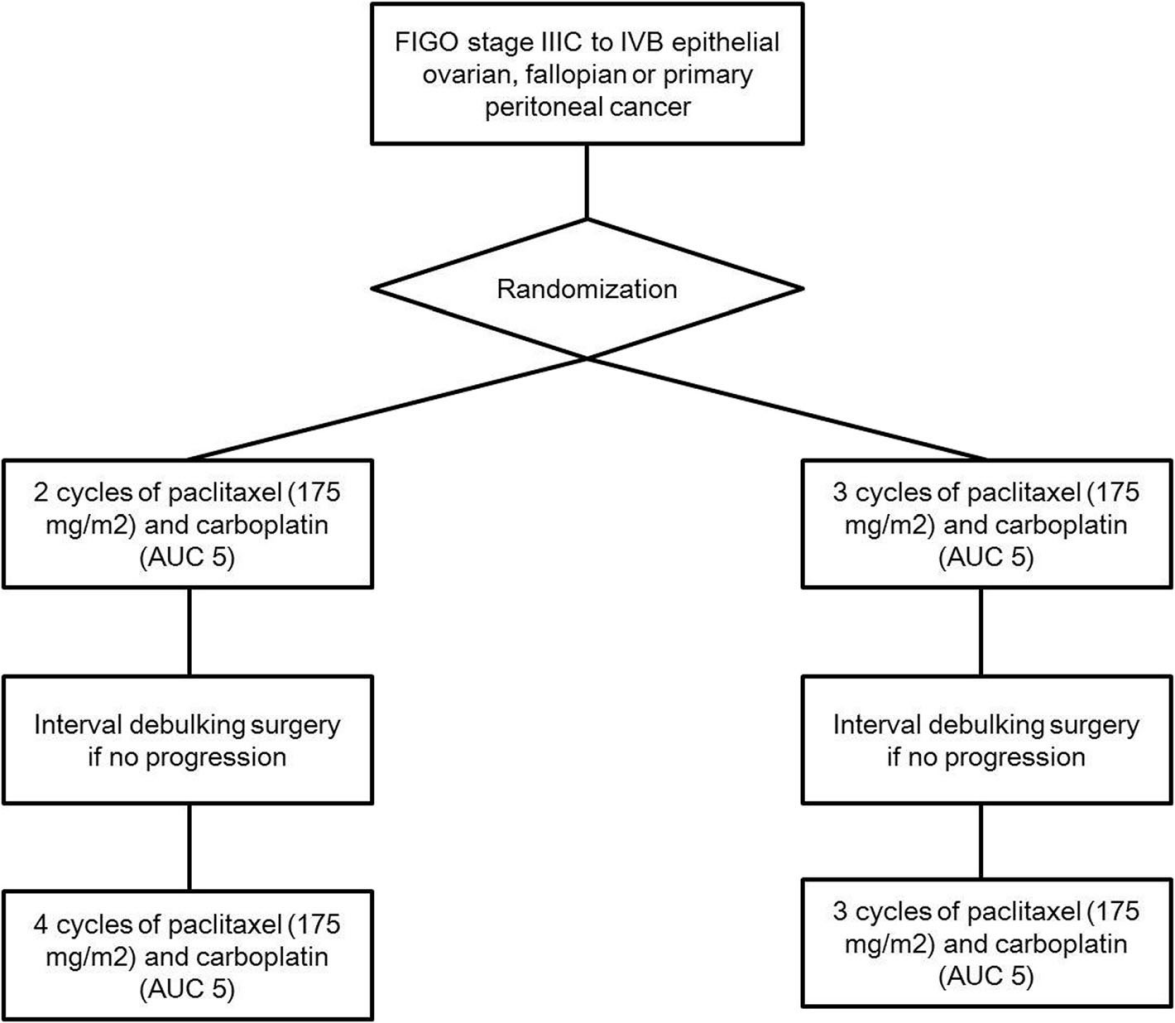
The diagram for the current study

Fig. 1 shows the diagram for this study. Patients in the control group will receive three cycles of NAC using paclitaxel (175 mg/m^2^) and carboplatin (area under curve, AUC; 5.0) every 3 weeks. We will perform IDS 3 weeks after the last cycle of NAC and within 6 weeks at maximum if there is no evidence of disease progression. Then, adjuvant chemotherapy with the same regimen and dose will be started 6 weeks after IDS and within 6 weeks. Adjuvant chemotherapy will be administered up to three cycles.

On the experimental arm, patients will receive two cycles of NAC using paclitaxel (175 mg/m^2^) and carboplatin (AUC; 5.0) every 3 weeks. IDS will be conducted 3 weeks after the last cycle of NAC and within 6 weeks if there is no evidence of disease progression, and then we will start adjuvant chemotherapy with the same regimen and dose 3 weeks after IDS and within 6 weeks after it. Adjuvant chemotherapy will be given up to four cycles.

Moreover, additional chemotherapy will be given up to three cycles depending on the discretion of principle investigators if there are residual tumours in both groups. In patients with disease progression after NAC, IDS will be cancelled, but patients will still be followed up during this study. If patients show grade 3 or 4 hematologic toxicity based on Common Terminology Criteria for Adverse Events (CTCAE) version 5.0 [14], we will gradually reduce doses of paclitaxel and carboplatin according to our protocol as follows: level 1, 20% dose reduction of paclitaxel and carboplatin; level 2, 40% dose reduction of paclitaxel and carboplatin.

In terms of IDS, maximal debulking resection of all visible lesions will be performed, and the size of residual tumour after IDS will be classified as follows: R0, no visible residual tumour; R1, ≤0.5 cm; R2 ≤ 1 cm; R3 > 1 cm. All procedures will be recorded on the Korean Gynecologic Oncology Group operation protocol [15], Fagotti score [16] and Sugarbaker’s Peritoneal Cancer Index (PCI) forms [17].

### Assessment of outcomes

#### End points

The primary end point is PFS, which is defined as the days from randomization to disease relapse or progression or death of other diseases defined as the last day when the patients are alive without any evidence of disease recurrence.

The secondary end points include effect and safety variables based on the number of cycles of NAC. As effect variables, time to tumour progression (TTP), OS, success rate of optimal cytoreduction, tumour response after NAC, IDS and adjuvant chemotherapy, radiologic investigation after IDS, tumour response by positron emission tomography-computed tomography (PET-CT) after neoadjuvant chemotherapy and quality of life will be evaluated. TTP is defined as the days from randomization to disease relapse or progression or death related with this disease, and OS means the days from randomization to death from any cause defined as the last day when the patients are alive. Tumour response after chemotherapy will be evaluated according to the new response evaluation criteria in solid tumours guideline (version 1.1) [18], and we will investigate tumour response after IDS based on the criteria suggested in European Organization for Research and Treatment of Cancer (EORTC) 55,971 trial [4]. Furthermore, we will assess quality of life with the EORTC Quality of Life Questionnaire-Core Questionnaire (EORTC QLQ-C30), the EORTC QLQ-Ovarian Cancer Module, the Functional Assessment of Cancer Therapy-Ovarian Cancer and the EuroQol five-dimension five-level questionnaire.

In terms of safety variables, we will evaluate adverse events, surgical complexity, postoperative complications and safety of IDS. Adverse events will be assessed by CTCAE version 5.0 [14], and surgical complexity and postoperative complications will be investigated using the Surgical Complexity Scoring system [19], and the Memorial Sloan Kettering Cancer Center Surgical Secondary Events grading system [20].

#### Follow-up

Table 1 shows the schematic diagram for the schedule in this study. Informed consent, randomization considering the inclusion and exclusion criteria, physical examination, WHO performance status, three tumour markers (CA-125, CEA and human epididymis protein 4), status of BRCA mutation, computed tomography (CT), PET-CT and quality of life will be checked at the screening time. WHO performance status, the three tumour markers and adverse events will be assessed in each cycle of chemotherapy, and CT will be conducted before and after IDS and after the sixth cycle of chemotherapy, whereas PET-CT will be checked before IDS. Postoperative complications will be checked after IDS, and quality of life will be added after IDS, after the sixth cycle of chemotherapy, and 6 months of the completion of chemotherapy. Moreover, we will follow up all enrolled patients every 3 months for the first 2 years and then every 6 months for the next 3 years with CT and tumour markers.

**Table 1:** Schematic diagrams for the schedule

	Screening	1st cycle	2nd cycle	3rd cycle	IDS	4th cycle	5th cycle	6th cycle	Follow-up
(1) Control group									
Informed consent	O								
Review of the inclusion and exclusion criteria	O								
WHO performance status & physical examination	O	O	O	O	O	O	O	O	
Tumour markers^1^	O	O^2^	O^2^	O^2^	O^3^	O^2^	O^2^	O^4^	O^5^
BRCA test	△^6^								
Tumour measurements (CT, PET-CT)	O^7^			O^7^	O^8^			O^8^	O^9^
Adverse events		O	O	O		O	O	O	
Postoperative complications					O				
Quality of life	O^10^				O^11^			O^11^	O^12^
	Screening	1st cycle	2nd cycle	IDS	3rd cycle	4th cycle	5th cycle	6th cycle	Follow-up
(2) Experimental group									
Informed consent	O								
Review of the inclusion and exclusion criteria	O								
WHO performance status & physical examination	O	O	O	O	O	O	O	O	
Tumour markers^1^	O	O^2^	O^2^	O^3^	O^2^	O^2^	O^2^	O^4^	O^5^
BRCA test	△^6^								
Tumour measurements (CT, PET-CT)	O^7^		O^7^	O^8^				O^8^	O^9^
Adverse events		O	O		O	O	O	O	
Postoperative complications				O					
Quality of life	O^10^			O^11^				O^11^	O^12^

^1^Cancer antigen-125 (CA-125), carcinoembryonic antigen (CEA), human epididymis protein-4 (HE-4), premenopausal Risk of Ovarian Malignancy Algorithm (ROMA), postmenopausal ROMA

^2^Performed within one week before the onset of chemotherapy

^3^Performed within one week before interval debulking surgery (IDS)

^4^Peformed within one week before the onset of the sixth cycle of chemotherapy and three weeks its completion. If total number of chemotherapy cycles is seven or more, tumour markers are evaluated three weeks after the completion of the last cycle of chemotherapy

^5^Performed every three months for the first two years and then every six months for the last three years

^6^Performed selectively after informed consent (one additional cancer panel using tumour tissues can be performed after informed consent if there is disease recurrence or progression)

^7^Peformed at the screening time and after the completion of neoadjuvant chemotherapy

^8^Only CT is performed three weeks after IDS (within six weeks), and three weeks after the completion of the 6th cycle of chemotherapy. If total number of chemotherapy cycles is seven or more, CT is performed three weeks after the completion of the last cycle of chemotherapy

^9^Only CT is performed every three months for the first two years and then every six months for the last three years

^10^EORTC QLQ-C30, EORTC QLQ-Ov28, FACT-O, EQ-5D-5 L are administered to evaluate quality of life

^11^EORTC QLQ-C30, EORTC QLQ-Ov28, FACT-O, EQ-5D-5 L, CTSQ are administered after IDS and three weeks after the completion of the sixth cycle of chemotherapy

^12^EORTC QLQ-C30, EORTC QLQ-Ov28, FACT-O, EQ-5D-5 L are administered six months after the completion of the sixth cycle of chemotherapy

#### Interim analysis and monitoring

We designed this study without an interim analysis, and therefore the sample size will not be changed during the study protocol. Data and Safety Monitoring Committee will monitor data every 6 months; committee members will not participate in this study and have no independent conflicts of interest.

#### Statistical analysis

We will conduct statistical analyses in both per-protocol (PP) and intention-to-treat (ITT) groups. Patients in the ITT groups will be assigned randomly without violating the inclusion and exclusion criteria, whereas the PP groups will comprise patients who complete the planned treatment without any of the following violations: failure to complete the planned NAC, IDS and adjuvant chemotherapy; failure to complete tumour evaluation; usage of onco-thermia, HIPEC or herbal medicine that can affect prognosis during the treatment.

Survival analyses will be conducted by the Kaplan-Meier method with the log-rank test, and independent prognostic factors will be identified using the Cox proportional hazard model. Dichotomous variables will be analysed by chi-square or Fisher’s exact test, whereas continuous variables will be compared with Student’s T or Mann-Whitney U test.

## Discussion

Although NAC is already used in clinical settings for advanced ovarian, fallopian, and primary peritoneal cancer, there is a lack of studies that address the most appropriate number of NAC cycles. Theoretically, NAC can help in reducing postoperative complications and increasing the success rate of optimal cytoreduction, which affects prognosis, but tumours hidden after NAC may not be removed during IDS and can thereby act as a focus of disease recurrence [12]. For this study, we hypothesize that reducing one cycle of NAC may contribute to more cytoreduction improving prognosis, as shown in a previous trial [13].

When we analysed the results from four RCTs with NAC [4–7], four and three cycles increased the rate of optimal cytoreduction by 45 and 35%, respectively, compared with PDS. As a result of these findings, we can expect that reducing one cycle of NAC may lead to a 10% decrease in optimal cytoreduction. Inversely, more excision of visible tumours after reducing one cycle of NAC might increase survival under the assumption that total cycles of chemotherapy are the same.

However, reducing one cycle of NAC can increase complications after IDS because of more aggressive resection of tumours. In the four RCTs, the postoperative complication rates were 24% in PDS and 10 and 4.6%, respectively, for three and four cycles of NAC. These findings suggest that reducing one cycle of NAC can lead to a 5% increase in postoperative complications, which may be clinically acceptable, and that two cycles of NAC can be more favourable for improving clinical outcomes of patients with advanced ovarian, fallopian or primary peritoneal cancer.

Although IDS after one cycle of NAC can also potentially improve prognosis, its application in clinical settings or trials seems to be premature because of the lack of experience or knowledge about the efficacy and safety of single-course NAC. However, the reduction of one cycle, from three cycles to two, maybe reasonable in clinical trials. If two cycles of NAC are superior to three cycles in this study, we can expect prolonged survival by more tumour cytoreduction with just two cycles, which then could replace HIPEC’s high cost and related renal and hepatic complications (4–7%) for improving survival [13]. Even if the prognosis is not different between three and two cycles of NAC in this study, this result will support the rationale that three cycles of NAC may be adequate for patients with advanced ovarian, fallopian or primary peritoneal cancer.

### Trial registration

The current study was registered at ClinicalTrials.gov (NCT03693248) on October 2, 2018.

## Data Availability

Not applicable.

## Abbreviations

AUC: Area under curve
CA-125: Cancer antigen 125
CEA: Carcinoembryonic antigen
CT: Computed tomography
CTCAE: Common terminology criteria for adverse events
EORTC: European Organization for research and treatment of cancer
EORTC QLQ-C30: EORTC quality of life questionnaire-core questionnaire
EORTC QLQ-OV28: EORTC QLQ-ovarian cancer module
EQ-5D-5 L: EuroQol five-dimension five-level questionnaire
FACT-O: Functional assessment of cancer therapy-ovarian cancer
FIGO: International federation of gynecology and obstetrics
HIPEC: hyperthermic intraperitoneal chemotherapy
IDS: Interval debulking surgery
ITT: Intention-to-treat
NAC: Neoadjuvant chemotherapy
PDS: Primary debulking surgery
PP: Per-protocol
OS: Overall survival
PET-CT: Positron emission tomography-computed tomography
PFS: Progression-free survival
TTP: Time to tumor progression
WHO: World health organization
